# Association between vitamin A and D levels and the risk of type 1 diabetes mellitus in children

**DOI:** 10.3389/fnut.2026.1837627

**Published:** 2026-06-10

**Authors:** Shuhan Rao, Tiewei Li, Zhaoli Yan, Xiaojuan Li, Wan Sun, Shengnan Li, Ligong Hou

**Affiliations:** 1 Department of Clinical Laboratory, Zhengzhou Key Laboratory of Children’s Infection and Immunity, Children’s Hospital Affiliated to Zhengzhou University, Zhengzhou, China; 2 Henan International Joint Laboratory of Prevention and Treatment of Pediatric Diseases, Children’s Hospital Affiliated to Zhengzhou University, Zhengzhou, China; 3 Department of Endocrinology, Affiliated Hospital of Inner Mongolia Medical University, Inner Mongolia Medical University, Hohhot, China

**Keywords:** children, relevance, type 1 diabetes mellitus, vitamin A, vitamin D

## Abstract

**Background:**

Vitamin A (VA) and vitamin D (VD) play crucial roles in immune regulation, yet their status in pediatric type 1 diabetes mellitus (T1DM) remains underexplored. This study aims to investigate the association between VA and VD levels and T1DM in children, providing a scientific basis for potential nutritional intervention strategies.

**Methods:**

This retrospective observational study was conducted at the Children’s Hospital Affiliated to Zhengzhou University. The study enrolled 31 children diagnosed with T1DM who underwent vitamin testing between January 2022 and October 2025. A control group of 31 children who received health examinations and VA assessments during the same period was included for comparison. Clinical and laboratory data were extracted from the hospital’s electronic medical records system.

**Results:**

The levels of serum VA and VD in children with T1DM were significantly lower than those in the control group, and the deficiency rate was correspondingly increased. Correlation analysis showed that the levels of VA (*r* = −0.708, *p* < 0.001) and VD (*r* = −0.366, *p* = 0.003) were negatively correlated with fasting blood glucose. Multivariate regression analysis identified VA (OR = 0.974, 95% CI: 0.959–0.990, *p* = 0.001) and VD (OR = 0.857, 95% CI: 0.761–0.965, *p* = 0.011) as independently associated factors for T1DM after adjustment for body temperature, heart rate, respiratory rate and BMI. Further analysis found an association between VA and VD deficiency and T1DM prevalence status in children.

**Conclusion:**

Serum levels of VA and VD are significantly associated with fasting blood glucose in children and show an independent association with T1DM. These findings suggest that maintaining appropriate VA and VD status may have some significance in the clinical management of T1DM in children, but they still need to be verified by rigorous longitudinal and interventional studies.

## Introduction

Type 1 diabetes mellitus (T1DM) is a chronic autoimmune disease characterized by polydipsia, polyphagia, polyuria, and weight loss. It accounts for approximately 90% of diabetes cases in children and adolescents (1). Globally, the incidence of T1DM has increased by an estimated 2–3% annually over recent decades (2). In China, the incidence of T1DM in children under 15 years old has almost quadrupled in the past two decades, and the number of patients has become the fourth highest in the world, making it an important part of chronic diseases in children and adolescents (3, 4). T1DM significantly impacts the growth and development of children and adolescents. It can disrupt musculoskeletal development, the puberty process, and peak bone mass formation, seriously affecting the physical and mental health of children (5). Owing to their young age at onset and limited capacity for self-care and self-management, children with T1DM are particularly vulnerable to suboptimal glycemic control. This predisposes them to both acute and chronic complications, ultimately compromising their long-term quality of life and survival (6, 7). T1DM is the result of the interaction between genetic predisposition, immune system disorders, and external environmental factors (8). As a key environmental regulatory factor, dietary nutrition is not only the material basis for children’s growth and development, but also may play a key role in the occurrence and development of T1DM.

Vitamin A (VA) and vitamin D (VD) are essential fat-soluble micronutrients that play important roles in immune regulation (9). As a dietary antioxidant, VA can prevent the development of autoimmunity by inhibiting the accumulation of reactive oxygen species and regulating the immune response (10). VD can participate in the occurrence and development of diabetes by regulating immune and inflammatory responses and promoting insulin synthesis and secretion (11). In a spontaneous mouse model of T1DM, a high-VA diet has been shown to reduce diabetes incidence by attenuating immune cell infiltration into pancreatic islets (12). A number of studies have shown that VD deficiency is common in patients with T1DM, VD supplementation can effectively reduce the risk of T1DM, protect islet *β*-cell function, and help control blood glucose (13–17). Most of the existing studies focus on adults or type 2 diabetes. However, the pathogenesis of T1DM in children is fundamentally different from that of type 2 diabetes, and the dose requirements of VA and VD and metabolic efficiency in children are significantly different from those in adults. Therefore, this study aimed to investigate the association between VA and VD levels and T1DM in children.

## Materials and methods

### Study design and population

This study was a retrospective case–control study conducted at the Children’s Hospital Affiliated to Zhengzhou University. The T1DM consisted of 31 hospitalized children diagnosed with T1DM from January 2022 to October 2025, who voluntarily underwent vitamin testing. The inclusion criteria for the T1DM group in this study are as follows: (1) Age between 3 and 14 years; (2) Meet the diagnostic criteria of “Expert Consensus on Standardized Diagnosis and Treatment of T1DM in Children in China (2020 Edition)” (18). That is, diabetes can be diagnosed if it meets one of the following four items: (1) Fasting blood sugar ≥7.0 mmol/L; (2) Blood sugar ≥11.1 mmol/L 2 h after oral glucose tolerance load [glucose 1.75 g/kg (body weight), maximum glucose 75 g]; (3) HbA1c ≥ 6.5%; (4) Random blood sugar ≥11.1 mmol/L accompanied by symptoms and signs of diabetes; (3) Have complete medical records. To ensure comparability, 31 children with normal physical examinations during the same period, whose age and gender were matched with those in the case group, were selected as the control group according to the principle of 1:1 group design. The inclusion criteria for the control group in this study are as follows: (1) Age between 3 and 14 years; (2) Good health status without complications; (3) Possessing complete clinical data and laboratory test data related to this study. To ensure the homogeneity of the study population and reduce confounding factors, the following exclusion criteria were adopted: (1) The final diagnosis was type 2 diabetes or other types of diabetes; (2) Those suffering from congenital diseases, autoimmune diseases, and hematological diseases; (3) Those suffering from chronic diseases such as pulmonary tuberculosis, asthma, gastrointestinal diseases, and severe liver and kidney diseases; (4) Those suffering from short stature, obesity, developmental delay, Cushing’s syndrome, thyroid disease, and other endocrine diseases; (5) Had received routine supplementation of VA and VD before admission.

This study adheres to the policies of the Declaration of Helsinki and has been approved by the Ethical Review Committee of Henan Children’s Hospital (2025-IITGC-034). All procedures, including the vitamin level assessments which were performed at the request of the patients or their guardians as part of clinical care, were part of routine practice. Data collection is retrospective, and strict anonymization protocols are followed to ensure confidentiality. As this study is a retrospective one, the Ethics Review Board waived the requirement for informed consent (2025-IITGC-034).

### Data collection

Clinical data including age, gender, body temperature, BMI, respiratory rate, and heart rate, along with laboratory test indicators such as VA, VD, fasting blood glucose (FBG), alanine aminotransferase (ALT), aspartate aminotransferase (AST), creatinine (Cr), uric acid (UA), glycosylated hemoglobin (HbA1c), C-peptide (C-PR), etc., were extracted from the hospital’s electronic medical records. All these indicators were the fasting test results of children in both groups within 24 h after admission. ALT, AST, Cr, and UA were all measured using an automated Beckman biochemical analyzer (Beckman Coulter, California). HbA1c and C-PR were all measured using an automated Roche electrochemiluminescence analyzer (Roche Diagnostics, Germany).

### Measurement of VA and VD

Serum VA and VD were detected using the ultra-high performance liquid chromatography–tandem mass spectrometry analysis system (Waters Xevo TQ-S Micro, Waters Corporation, Massachusetts). The sample pretreatment process follows the instructions of the commercial reagent kit for VA and VD (Shanghai Kehua Bio-Engineering Co., Ltd. China), and 5 μL of the sample was injected for analysis. The electric spray ionization source is used for positive mode collection. The capillary voltage is 3.0kv, the ion source temperature is 150 °C, the desolvent temperature is 500 °C, the desolvent gas is 1,000 L/Hr, and the cone hole gas is 50 L/Hr. Use Masslynx software (Waters Corporation, Massachusetts) to complete data collection and qualitative and quantitative analysis. According to the Expert Consensus on Clinical Application of VA and VD in Children in China (2024), VA deficiency is defined as a level below 200 ng/mL, and VD deficiency is defined as a level below 12 ng/mL.

### Statistical analysis

All statistical analyses were completed using SPSS 25.0. All statistical indicators were tested for normality and homogeneity of variance. When the measurement data conform to the normal distribution, the mean ± standard deviation (SD) is used for description, and the data between the two groups are compared using two independent sample *t*-tests. When the measurement data do not conform to the normal distribution, the median (inter-quartile range) [M (P25, P75)] is used for description, and the data between groups are compared using non-parametric tests. For counting data, frequency (*n*) and percentage (%) are used for description, and the Chi-square test (*χ*^2^) is used to compare the rates between groups. Spearman correlation analysis was used to assess the relationship between VA and VD and blood sugar and other clinical and laboratory parameters. Multivariate logistic regression analysis was performed to determine whether VA and VD were independent risk factors for T1DM. Variables with *p*-values below 0.05 in the univariate logistic analysis were included in the multivariate logistic regression model. Using *α* = 0.05 as the test level, *p* < 0.05 is considered to be statistically significant.

## Results

### Study population characteristics

This study included 31 children with T1DM and 31 healthy children. Children with T1DM are defined as the T1DM group, while healthy children are classified as the control group. The basic characteristics of the control group and the T1DM group are summarized in Table 1. There was no significant difference in age and gender composition between the T1DM group and the control group (*p* > 0.05), indicating comparability. The body temperature, respiratory rate, and heart rate in the T1DM group were higher than those in the control group, and the BMI was lower than that in the control group, with statistical significance (*p* < 0.05). There was a statistically significant difference in living types between the two groups (*p* < 0.001). The proportion of rural living in the T1DM group (29.0%) was higher than that in the control group (19.4%).

**Table 1:** Basic characteristics of study participants in control and diabetic groups.

Variables	Control (*n* = 31)	T1DM (*n* = 31)	*p*
Age (years)	7.2 (5.2, 10.0)	7.6 (5.3, 10.0)	0.878
Gender			1
Male, *n* (%)	14 (45.2%)	14 (45.2%)	
Female, *n* (%)	17 (54.8%)	17 (54.8%)	
Temperature (°C)	36.4 (36.2, 36.5)	36.5 (36.4, 36.7)	0.008
Respiratory rate (rate/min)	20 (19, 22)	23 (22, 26)	<0.001
Heart rate (rate/min)	94 (83, 99)	100 (92, 114)	0.004
BMI (kg/m^2^)	15.66 (14.51, 17.90)	14.89 (13.77, 15.44)	0.01
FBG (mmol/L)	4.57 (4.33, 4.80)	16.41 (10.90, 20.03)	<0.001
ALT (U/L)	12. 3 (10.8, 14.0)	16.1 (12.0, 22.1)	0.023
AST (U/L)	23.8 (19.8, 28.8)	23.5 (18.0, 31.1)	0.627
Cr (umol/L)	35.6 (29.1, 38.5)	32 (28.9, 39.3)	0.735
UA (umol/L)	256.3 (237.3, 297.7)	245.4 (155.3, 345.3)	0.379
HbA1c (%)	/	12.45 (9.73, 13.93)	/
C-PR (ng/mL)	/	0.46 (0.24, 0.59)	/
Residential classification			<0.001
Urban, *n* (%)	25 (80.6%)	22 (71.0%)	
Rural, *n* (%)	6 (19.4%)	9 (29.0%)	

FBG, fasting blood-glucose; ALT, alanine aminotransferase; AST, aspartate aminotransferase; Cr, creatinine; UA, uric acid; HbA1c, glycosylated hemoglobin; C-PR, C-peptide.

### VA, VD levels and nutritional status in two groups of children

According to the “Expert Consensus on Clinical Application of VA and VD in Children in China (2024)” (19), the two groups of children were classified into the VA deficiency group (<200 ng/mL), the non-VA deficiency group (≥200 ng/mL), the VD deficiency group (<12 ng/mL), and the non-VD sufficiency group (≥12 ng/mL) based on their VA and VD levels. Analysis of the serum VA and VD levels of children in the two groups revealed that the concentrations of VA and VD in the T1DM group were significantly lower than those in the control group (Table 2). Compared with the control group, the children with T1DM had significantly higher prevalence rates of VA deficiency (51.6% vs. 3.2%, *p* < 0.001) and VD deficiency (35.5% vs. 3.2%, *p* < 0.001). At the same time, in the children with type 1 diabetes, the non-VA deficiency group and the non-VD deficiency group both accounted for 96.8%. In the control group, the non-VA deficiency group and the non-VD deficiency group accounted for 48.4 and 64.5%, respectively.

**Table 2:** VA, VD levels and nutritional status in two groups of children.

Variables	Control (*n* = 31)	T1DM (*n* = 31)	*p*
VA (ng/ml)	363.50 (318.93, 407.17)	185.38 (102.44, 242.46)	<0.001
VD (ng/ml)	18.76 (14.20, 25.12)	14.18 (9.40, 19.21)	0.013
VA status			<0.001
Non-VA deficiency, *n* (%)	30 (96.8%)	15 (48.4%)	
VA deficiency, *n* (%)	1 (3.2%)	16 (51.6%)	
VD status			<0.001
Non-VD deficiency, *n* (%)	30 (96.8%)	20 (64.5%)	
VD deficiency, *n* (%)	1 (3.2%)	11 (35.5%)	

VA, vitamin A; VD, vitamin D.

### Correlation between VA and VD levels and clinical indicators

The relationship between VA, VD, and clinical indicators was further explored (Table 3). VA was negatively correlated with body temperature (*r* = −0.296, *p* = 0.019), respiratory rate (*r* = −0.409, *p* = 0.001), heart rate (*r* = −0.334, *p* = 0.008), and FBG (*r* = −0.708, *p* < 0.001). There was no significant correlation between VA and age, BMI, ALT, AST, UA, and Cr. VD was negatively correlated with age (*r* = −0.327, *p* = 0.010), FBG (*r* = −0.366, *p* = 0.003), and Cr (*r* = −0.347, *p* = 0.006), and positively correlated with ALT (*r* = 0.254, *p* = 0.046). VD was not correlated with body temperature, respiratory rate, heart rate, BMI, AST, and UA.

**Table 3:** Correlation analysis of VA, VD levels and clinical indicators.

Variables	VA		VD	
	*r*	*p*	*r*	*p*
Age (years)	−0.101	0.435	−0.327	0.010
Temperature (°C)	−0.296	0.019	0.031	0.814
Respiratory rate (rate/min)	−0.409	0.001	−0.196	0.126
Heart rate (rate/min)	−0.334	0.008	0.006	0.963
BMI (kg/m^2^)	0.177	0.170	−0.147	0.253
FBG (mmol/L)	−0.708	< 0.001	−0.366	0.003
ALT (U/L)	0.044	0.734	0.254	0.046
AST (U/L)	−0.239	0.061	−0.018	0.893
Cr (umol/L)	−0.069	0.592	−0.347	0.006
UA (umol/L)	0.086	0.505	0.249	0.051

FBG, fasting blood-glucose; ALT, glutamic-pyruvic transaminase; AST, glutamic oxalacetic transaminase; Cr, creatinine; UA, uric acid; VA, vitamin A; VD, vitamin D.

### Relationship between vitamin levels, nutritional status and T1DM

Multivariate binary logistic regression analysis was used to evaluate whether VA and VD were independent related factors for T1DM. Variables with *p* < 0.05 in the univariate regression analysis were incorporated into the multivariate binary logistic regression analysis, including body temperature, respiratory rate, heart rate and BMI. As presented in Table 4, multivariate regression analysis confirmed that VA (OR = 0.974, 95% CI: 0.959–0.990, *p* = 0.001) and VD (OR = 0.857, 95% CI: 0.761–0.965, *p* = 0.011) were independently associated with T1DM after adjusting for the aforementioned variables. Further analysis found an association between VA and VD deficiency and T1DM prevalence status in children.

**Table 4:** Relationship between vitamin levels, nutritional status and T1DM.

Variables	Univariate		Multivariate	
	OR (95%CI)	*p*	OR (95%CI)	*p*
VA (ng/ml)	0.976 (0.965–0.987)	<0.001	0.974 (0.959–0.990)	0.001
VD (ng/ml)	0.910 (0.842–0.984)	0.019	0.857 (0.761–0.965)	0.011
VA status				
Non-VA deficiency, *n* (%)	1		1	
VA deficiency, *n* (%)	32.000 (3.867–264.795)	0.001	25.314 (2.152–297.721)	0.010
VD status				
Non-VD deficiency, *n* (%)	1		1	
VD deficiency, *n* (%)	16.500 (1.973–137.996)	0.010	14.054 (1.220–161.935)	0.034

Adjust according to body temperature, breathing rate, heart rate and BMI. VA, vitamin A; VD, vitamin D.

## Discussion

T1DM is a typical T-cell-mediated autoimmune disease (20). Its core pathological feature is the progressive destruction of pancreatic *β* cells by immune-mediated mechanisms, ultimately leading to absolute insulin deficiency, and the patients need to rely on exogenous insulin replacement therapy for life (5). During the immune effector stage, multiple immune cells and cytokines jointly participate in the destruction of β cells. Macrophages, once activated, secrete pro-inflammatory cytokines such as tumor necrosis factor (TNF-*α*) and interleukin-1β (IL-1β), which directly exert cytotoxic effects on β cells (21–23). The production of these pro-inflammatory cytokines, in turn, induces the expression of chemokines and adhesion molecules, thus promoting the recruitment of lymphocytes and macrophages to the pancreas (24, 25). VD can inhibit the production of chemokines, reduce the over-expression of receptors on the surface of monocytes, and prevent the abnormal activation of receptors, thereby avoiding severe inflammation (26). Meanwhile, it regulates the maturation and function of macrophages and antigen—presenting cells, induces the formation of dendritic cell immune tolerance phenotypes, weakens their stimulatory effect on specific T cells, and reduces their ability to initiate immune responses (26). CD8^+^ cytotoxic T cells can directly kill *β* cells by recognizing MHC I molecules on the surface of β cells (27, 28). VD can reduce the expression of MHC I molecules on the surface of pancreatic β cells, directly decreasing the probability of their recognition and attack by immune cells, and reducing cell apoptosis (29). CD4^+^ helper T cells differentiate into subtypes such as Th1 and Th17, secrete pro-inflammatory cytokines, further amplifying the inflammatory response and inhibiting β cell insulin synthesis and secretion (30). VD can inhibit the proliferation and activation of CD4^+^ cells, reduce the release of pro-inflammatory cytokines, promote the differentiation of regulatory T cells and IL-10 secretion, induce the apoptosis of autoreactive T cells, and balance the immune response (26, 31, 32). An imbalance in the Th1/Th2 axis represents a pivotal mechanism in the pathogenesis of T1DM (33). In this state, an overactivation of Th1 cells leads to increased secretion of interferon-gamma (IFN-*γ*), which enhances antigen presentation and heightens the susceptibility of *β*-cells to immune-mediated attack (34). Concurrently, the protective, anti-inflammatory functions mediated by Th2 cells are suppressed (34). VA may counteract this pathogenic imbalance by modulating T-helper cell differentiation. Specifically, supplementation with VA or its active metabolite, retinoic acid (RA), has been shown to inhibit Th1 cell activation and IFN-γ production while promoting Th2 cell development and the expression of interleukin-10 (IL-10) (35–37). This shift in the Th1/Th2 balance reduces the autoimmune attack on *β*-cells, thereby potentially decreasing the risk of developing T1DM.

VA plays an indispensable role in various physiological processes, such as visual protection, growth and development, cell differentiation and proliferation, and immune regulation. Its physiological activity is mainly achieved through the metabolite RA (38). In recent years, an increasing number of studies have shown that VA not only regulates immune imbalance and reduces inflammatory responses but also participates in the regulation of T1DM onset by protecting beta cells, thereby influencing disease progression and the occurrence of complications. This provides a new research direction for the prevention and adjunctive treatment of T1DM. Specifically, VA and its metabolites are key regulatory factors for maintaining the normal structure of the pancreas and the function of beta cells (39–41). Animal experiments have shown that mice fed with VA-deficient diets have significantly lower body weight and pancreatic weight than those with adequate VA intake, and they exhibit problems such as impaired glucose tolerance, reduced insulin secretion after glucose stimulation, and decreased staining intensity of insulin in pancreatic islets (42). This result suggests that VA deficiency directly damages the function of beta cells. On this basis, studies on different T1DM animal models further verified the protective effect of VA. In streptozotocin (STZ) or alloxan-induced T1DM models, pre-administration of palmitic acid retinyl ester can protect beta cells from damage and significantly delay the disease onset process (43). In the STZ-induced diabetes mouse model, it was also found that retinoic acid (RA) can improve the vascularization of pancreatic islets in diabetic mice and alleviate hyperglycemic symptoms by enhancing insulin gene expression in beta cells and inhibiting beta cell apoptosis (44). While in VA-deficient rats, supplementing VA or administering retinoic acid treatment can maintain normal glucose-induced insulin secretion in pancreatic islets (45). At the same time, both animal experiments and human studies have confirmed that there is a significant VA metabolic disorder in T1DM. In animal models, the serum VA levels and liver retinol palmitate (VA storage form) levels of STZ-induced diabetic rats and BB rats (spontaneous T1DM model) are significantly lower than those of the normal control group (46–48). In human studies, compared with healthy individuals, the circulating VA levels, retinol binding protein (RBP) levels, and transthyretin (TTR) levels of T1DM patients are significantly lower, and this decrease is negatively correlated with glycemic control levels (such as HbA1c) and the degree of beta cell dysfunction (46, 49–52). It is worth noting that in this study, we also observed that the VA levels in children with T1DM were significantly lower and the deficiency rate was significantly higher, further supporting the above conclusion. In addition, VA metabolic abnormalities are closely related to T1DM complications, with the associations between diabetes retinopathy and diabetic nephropathy being the most prominent. For example, 11-cis retinaldehyde, a metabolite of VA, is a key component of the visual photoreceptor (rhodopsin), and the level of 11-cis retinaldehyde in the eyes of STZ-induced diabetic rats is significantly reduced, leading to impaired synthesis of rhodopsin, which may be involved in the early pathological process of retinopathy (53). Clinical studies have also observed that the dietary retinol intake of patients with diabetic retinopathy is lower than that of patients without retinopathy, suggesting that VA deficiency may increase the risk of retinopathy (54). However, the association between the two is still controversial. A study in Belgium showed that the blood VA levels of T1DM children with retinopathy were higher than those of children without retinopathy (55). Apart from retinopathy, VA also plays an important role in T1DM-related kidney damage. Studies have shown that retinoic acid (RA) can alleviate the oxidative stress and inflammatory responses in the kidneys of STZ-induced diabetic rats by inhibiting the secretion of pro-inflammatory factors (such as TNF-*α*) and down-regulating the Toll-like receptor 4 (TLR4) signaling pathway (56–58). This, in turn, reduces the proteinuria level and improves diabetic nephropathy.

Apart from VA, VD is also closely related to the occurrence and development of T1DM. VD plays a crucial role in regulating the skeletal system, maintaining stable serum calcium and phosphorus levels, and immune regulation in the body. Relevant research results fully confirm that VD participates in the occurrence and development of T1DM through multiple pathways. It directly regulates insulin secretion and protects pancreatic *β* cells through immune regulation, and has preventive and auxiliary therapeutic value for T1DM (59). A case–control study by Changwei Liu et al. showed that serum VD levels were lower in T1DM patients than in controls (48.69 nmol/L vs. 57.93 nmol/L) (60). The VD deficiency rate was higher in the T1DM group than in the control group (13.18% vs. 5.76%) (60). In the present study, our data similarly showed that children with type 1 diabetes had lower serum VD levels than control children (14.18 ng/mL vs. 18.76 ng/mL) and a significantly higher prevalence of deficiency (35.5% vs. 3.2%). We speculate that the prevalence of VD deficiency in the T1DM group in this study was significantly higher than that in the study by Changwei Liu, possibly due to factors such as geographical distribution and detection season. Multiple meta-analyses and cross-sectional studies have further confirmed that the serum VD level of children with T1DM is significantly lower than that of healthy control groups, and the incidence of VD deficiency in children with T1DM is higher (14, 16, 59, 61–63). Based on this, related studies further explored the association between VD and the onset and treatment of T1DM. Epidemiological studies have shown that VD deficiency during infancy increases the risk of T1DM, and supplementing VD can reduce the risk (64, 65). In addition to preventing the onset, VD has also been widely studied in the treatment of T1DM. Gabbay’s study showed that T1DM patients who supplemented 2000 IU of VD daily for 18 months had a slower decline in C-peptide levels and a significant reduction in HbA1c levels within 6 months (66). Another 6-month randomized controlled trial also confirmed that supplementing VD can improve pancreatic *β*-cell function (67). Besides the impact on the onset and treatment of T1DM, VD levels are also closely related to chronic complications of T1DM, with the association with diabetic retinopathy being the most prominent. Wang Qian et al. found that low VD levels were associated with the severity of diabetic retinopathy (68). A Logistic regression analysis further confirmed that serum 25(OH)D deficiency was associated with the risk of proliferative diabetic retinopathy and was an independent risk factor for retinopathy in young T1DM patients (69). Apart from retinopathy, VD is also closely associated with T1DM-related kidney damage. Animal experiments showed that the 25(OH)D concentration in mice with diabetic nephropathy was significantly lower than that in the healthy control group, suggesting that VD may have a renal protective effect (70, 71). Further research found that low VD levels can predict an increased risk of progression of diabetic nephropathy or can be used as a predictive indicator for the severe progression of early diabetic nephropathy, and VD may be involved in the occurrence and development of early renal injury in T1DM patients (72, 73). From the above research results, it can be seen that children with T1DM generally have VD deficiency. Oral administration of VD can not only improve the residual pancreatic cell function and reduce the HbA1c level to effectively control blood sugar, but also has potential benefits in delaying microvascular complications such as diabetic retinopathy and diabetic nephropathy.

In the present study, we explored the association between VA and VD levels and T1DM in children. We found that children with T1DM had significantly lower VA and VD levels and higher deficiency rates than healthy children. The VA sufficiency rate was 96.8% in the control group and 48.4% in the T1DM group. The VD sufficiency rate was 96.8 and 64.5% in the control and T1DM groups, respectively. Correlation analysis showed that VA and VD levels were significantly correlated with fasting blood glucose. In addition, VA and VD were independently associated with T1DM in children after adjusting for confounding variables such as body temperature, respiratory rate, heart rate, and BMI. Further analysis found an association between VA and VD deficiency and T1DM prevalence status in children. This study is an observational case–control study, suggesting that the relationship between serum VA, VD levels and T1DM cannot establish a clear causal relationship. It is unclear whether preexisting vitamin deficiency contributes to the onset of T1DM through immune regulation or whether metabolic disorders and chronic inflammation of T1DM itself lead to depletion of vitamin reserves. This temporal uncertainty must be taken into account when interpreting the observed associations. Prospective longitudinal studies or Mendelian randomization approaches are needed to clarify the direction of causality.

This study has several limitations that should be carefully considered. First, this study was a single-center retrospective study with a small sample size, which may have introduced selection bias and limited statistical power. The highly inflated confidence intervals for VA deficiency (CI: 2.152–297.721), VD deficiency (CI:1.220–161.935) may be due to the extreme imbalance in the prevalence of vitamin deficiency between the T1DM and control groups. These wide confidence intervals indicate substantial statistical uncertainty, and the results should be interpreted with caution. Second, we did not account for important unmeasured confounders such as dietary vitamin intake, duration of sunshine, and time spent outdoors. Because this was a records-based retrospective study, precise data on individual dietary habits, duration of outdoor activities and sun exposure were not routinely recorded in medical records and could not be analyzed. In addition, we did not have HbA1c data for children in the control group, as this was not a physical examination item for healthy children at our center. Therefore, the potential confounding effect of glycemic control in the control group could not be assessed. Residual confounding by these unmeasured variables cannot be ruled out. Third, this retrospective study did not systematically record information on the course of the disease in children with T1DM, and vitamin status may vary with the duration of diagnosis and the level of long-term glycemic control. Long-term metabolic disorders may gradually depleting the body’s vitamin reserves. The current data from this study can only distinguish between children who are first visits and those who are not. However, due to the small sample size, the statistical power of these subgroup results is limited, and the conclusion’s credibility is not high. Finally, the effect of VA and VD supplementation on clinical outcomes in patients with diabetes was not addressed in this study. Future prospective studies with large samples are needed to collect detailed diet, lifestyle, and disease duration data and to more fully adjust for confounding factors (including season of blood sampling, duration of sunshine, and dietary vitamin intake) in all participants. In addition, attention should be paid to clearly distinguish between newly diagnosed and long-term T1DM children, and to analyze the changes in vitamin levels during the disease process.

## Conclusion

In conclusion, the data from this study indicate that children with T1DM have lower levels of VA and VD and a higher rate of deficiency. Correlation analysis showed that VA and VD levels were significantly correlated with FBG. Multivariate analysis showed that VA and VD were independently associated with T1DM, but retrospective studies could not determine causality. Whether optimizing VA and VD status contributes to the prevention or management of T1DM in children needs to be investigated through rigorous longitudinal and interventional studies.

## Data Availability

The raw data supporting the conclusions of this article will be made available by the authors, without undue reservation.
